# Guanxi HRM Practice and Employees’ Occupational Well-Being in China: A Multi-Level Psychological Process

**DOI:** 10.3390/ijerph17072403

**Published:** 2020-04-01

**Authors:** Jia Xu, Baoguo Xie, Bin Tang

**Affiliations:** 1 School of Political Science and Public Administration, Wuhan University, Wuhan 430072 China; jiaxu@whu.edu.cn (J.X.); bintangwhu@whu.edu.cn (B.T.); 2 School of Management, Wuhan University of Technology, Wuhan 430070, China

**Keywords:** guanxi HRM practice, psychological safety, occupational well-being, collectivistic culture

## Abstract

Chinese employees may experience and respond to guanxi human resource management (HRM) practice (e.g., recruiting, selecting, inducting and appraising employees based on personal relationships). Little has been done to examine the linkage between guanxi HRM practice and employees’ occupational well-being. This study investigates the psychological process of how guanxi HRM practice affects employees’ occupational well-being. The theoretical model of this study proposes that employee psychological safety mediates the relationship between guanxi HRM practice and occupational well-being, while collectivistic team culture moderates the relationship between guanxi HRM practice and psychological safety. Multi-level data from 297 employees nested within 42 teams support all hypotheses. This study reveals the cross-level effects of guanxi HRM practice and providing practical suggestions for future research on psychologically safe and healthy work environments.

## 1. Introduction

Over the past 40 years, occupational well-being has been gaining momentum as it has been realized to be a central concern not only for individual health but also for managers and organizations in terms of performance and overall productivity [1,2]. Many researchers have focused on the antecedents of occupational well-being and have found that psychosocial factors provided by the work environment are vital precursors of occupational well-being [3]. Nevertheless, little research has explored the relationship between human resource management and occupational well-being [4,5]. Human resource management (HRM) is a critical engine driver of organizational performance and sustainability which employees need to encounter every working day [6]. According to Peccei and van de Voorde [7], when HR practice is designed to provide the right mix of psychological job dimensions (e.g., optimal decision authority), work can improve occupational well-being. If HR practice is not designed in a supportive way (e.g., too much interpersonal pressure), work can trigger a stress reaction. It is necessary to examine the effect of HRM on occupational well-being. An investigation of the relationship between HRM and occupational well-being will facilitate both theoretical advancement and identification of managerial implication development.

Recent research that has focused on guanxi HRM practice has regarded it as the stressor of employee outcomes, which is defined as the extent to which HRM decisions are made based on personal relations [8]. Guanxi HRM practice is pervasive in contemporary Chinese organizations and has universal explanatory value in all organizational contexts [9]. Most empirical studies have shown that guanxi HRM practice involves a social dilemma that influences both positive and negative outcomes [10]. On the positive side, guanxi HRM practice can be beneficial for the parties involved, with more bonuses and promotion chances [11]. On the negative side, guanxi HRM practice can lead to decreased in-role and extra-role performance [12] and decreased trust in management [8]. However, few studies have examined the effect of guanxi HRM practice on employees’ occupational well-being. This lack of knowledge makes it difficult for Chinese managers to design and implement HRM in a way that promotes occupational well-being. Thus, the focus of the present study was to test the relationship between guanxi HRM practice and employees’ occupational well-being.

Furthermore, we still have much to learn about the “black box” of how guanxi HRM practice affects employees’ occupational well-being. Of the studies examining the influence of guanxi HRM practice on employee emotional exhaustion (e.g., Yang et al. [12]), few have explored the psychological mechanism governing this relationship. Guanxi HRM practice may stir employees’ psychological state of stress; thus, it can trigger individuals’ distrust and fear of being harmed or mistreated (e.g., Zhang et al., [13]). On this basis, we propose that psychological safety can be seen as a proxy of “resources loss,” which reflects employees’ affective reactions toward guanxi HRM practice. Therefore, following Zhang and colleagues [13], we integrate conservation of resources (COR) theory and psychological safety and examine the relationship between guanxi HRM practice and employees’ occupational well-being in the Chinese context. We attempt to reveal the mediating role of psychological safety in the relationship between guanxi HRM practice and employees’ occupational well-being.

Thirdly, it remains largely unanswered whether the negative effects of guanxi HRM practice emerge in all circumstances. Wu and Chaturvedi [14] provided evidence that the exact way that HRM contributes to employee outcomes depends on team culture. There is a need to align HRM practice with organizational culture in order to successfully implement HRM practice. In this study, we focus on collectivistic culture, as it plays a significant role in HR practice in China. Although collectivism was originally conceived at the societal level [15], previous research has shown large variation within a single culture [16]. We thus argue that team collectivistic culture can influence the effects of guanxi HRM practice, such that group collectivism moderates the relationship between employees’ perceptions of guanxi HRM practice and psychological safety.

**Figure 1:**
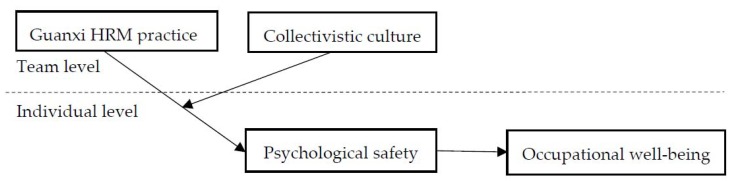
Theoretical Model.

The present study makes several contributions to the literature. First, to the best of our knowledge, few empirical studies have examined the effects of HRM on employees’ occupational well-being. We address this research gap by investigating one pervasive HRM style in the workplace of China: guanxi HRM practice, which may play an important role in connecting occupational well-being to that perceived by employees. Secondly, we identify employees’ perceptions of psychological safety as a key mediating mechanism that links guanxi HRM practice to occupational well-being. Finally, since there are unclear boundary conditions for the effects of guanxi HRM practice, we draw from the cultural value perspective to examine when guanxi HRM practice is related to employees’ perception, which, as a result, decreases employees’ psychological safety. The overall theoretical model is presented in Figure 1.

## 2. Literature Review and Hypothesis Development

### 2.1. Guanxi HRM Practice, Psychological Safety, Collectivistic Culture, and Occupational Well-Being

Guanxi, a Chinese term referring to mutually and reciprocal interpersonal connections, has been pervasive and extensively examined in China and the Chinese-language areas of Taiwan, Singapore, and other overseas Chinese communities for the last few centuries [17,18,19]. Guanxi has been found to be different from Western concepts of personal relationships (e.g., leader-member exchange), as it is characteristically long-term, involves unequal personal relationships, and is instrumentally purposive in nature. Guanxi can bring many benefits, such as reducing costs, bypassing or cutting out the bureaucratic maze, gaining information and privilege, and obtaining scarce resources [20]. Thus, guanxi is prominent in Chinese workplace relationships and has been considered one of the most important success factors in business, politics or everyday life [21]. Chinese companies adjust HR policies and practices to be compatible with the Chinese context and use guanxi HRM practice to win competitions [13].

Guanxi HRM practice refers to the degree to which HRM decisions, such as recruitment, task allocation, performance appraisal, promotion, and compensation, are influenced by personal relationships [22]. In other words, when team leaders make HRM decisions based on the quality of personal relationships with team members, they engage in guanxi HRM practice. Many studies have suggested that guanxi HRM practice serves as a double-edged sword [23]. On the one hand, guanxi HRM practice has been found to be positively related to desirable employee outcomes, such as career development [24]. One the other hand, some empirical research has suggested that guanxi HRM practice is unethical and has detrimental effects on employees’ trust in management [8], perceived fairness [25], and work engagement [26]. Despite this evidence, and other calls for future research on the mediators and moderators [27], surprisingly little is known about the influencing mechanism through which guanxi HRM practice is associated with employees’ occupational well-being.

Psychological safety is defined as “feeling able to show and employ one’s self without fear of negative consequences to self-image, status, or career” [28] (p. 708)—that is, whether an individual is confident to express true beliefs or ideas without worrying about negative consequences to career prospects. Bennis [29] argued that organizations must recognize and create a psychologically safe work environment, as safety is a fundamental human need. When employees feel a high level of psychological safety within a workgroup, they are likely to speak up and voice opinions [30], learning from failures [31]. Thus, psychological safety was found to be positively related to a host of employee outcomes, such as performance [32], creativity [33], helping behavior [34], and job satisfaction [35]. Research has explored four contextual factors for psychological safety: interpersonal relationships, group dynamics, leadership, and organizational norms [36]. Specifically, a supportive work context, positive leadership, and work design characteristics play an important role in shaping the psychological safety of employees [37].

Collectivism is typically conceptualized as the opposite of individualism, especially when contrasting East Asian and Western cultures. Within a workgroup, collectivistic culture presents a pattern of shared values, beliefs, and norms that emphasize interdependence, cooperation, and harmony between teams of employees [38]. Collectivistic culture typically influences the factors contributing to the development of interpersonal relationships [39]. In a collectivistic culture, individuals should assign priority to work relationships, and protecting harmonious relationships is important even if the fairness norms are violated. Employees in collectivistic cultures primarily view themselves as members of an extended organization rather than as individuals. As a result, they expect supervisors in their group to take care of them and place more emphasis on obligation and loyalty [40]. Accordingly, we believe that team collectivistic culture is likely to affect the attitudinal responses of employees to guanxi HRM practice.

Well-being is a multidimensional construct containing different types of well-being (occupational, psychological, spiritual) [41]. In the context of occupational health psychology, occupational well-being is defined as a positive assessment of one’s work life [42]. It is commonly reported in the literature that occupational well-being has both personal and organizational implications that require attention if organizations are to ensure a competitive advantage [43]. The relationship between occupational well-being and performance is well established [44,45]. Hence, there is a growing need to explore what leads to occupational well-being. Past research suggested that the main factors affecting occupational well-being include leadership [46], job demand [47], organizational support [48] and personal resources [49]. However, there has been limited research linking HRM to employees’ occupational well-being, even though there have been calls to focus on the effects of HRM on employee-centered outcomes, especially employee well-being [50].

### 2.2. Conservation of Resources (COR) Theory

As a resource-based theory of stress, conservation of resources (COR) theory provides a theoretical explanation of whether and how HRM practice impacts employee well-being. COR theory acknowledges the importance of resources for sustaining employee well-being. When these resources are threatened or lost, their loss may evoke distress, eventually resulting in decreased well-being [51]. In the context of the workplace, job resources are the aspects of work that initiate a motivational process. For example, decision authority and social support (supervisor, coworker, organization, customer, etc.) provide meaning to employees and satisfy their basic needs. Personal resources refer to individuals’ sense of their ability to control and impact their environment successfully. Personal resources such as optimism, psychological safety, self-esteem play a similar role as job resources [52]. It is pertinent for HR managers to design and deliver practices to help employees develop resources to cope with job demands and achieve goals.

According to the COR theory, when employees realize they do not have many resources, they will lose their enthusiasm and report more burnout and pressure, resulting in lower occupational well-being. Guanxi HRM practice as a work demand may threaten or deplete the individual’s valued resources. There is evidence that guanxi HRM practice is related to employee psychological conditions. For instance, Chen and O’Leary [27] found that guanxi HRM practice was negatively related to affective commitment. Thus, COR theory offers a theoretical explanation by linking guanxi HRM practice with the consumption of resources for minimizing employees’ occupational well-being.

### 2.3. Hypothesis Development

According to the COR theory, guanxi HRM practice can be viewed as a job demand as it exerts an energy-draining effect on employees through a stressful process that may undermine employees’ occupational well-being. On the one hand, guanxi HRM practice may decrease employees’ job resources, such as fairness perception. Guanxi HRM practice creates a work environment in which opaque promotion rules, informal exchange of opinions and sharing of valuable information, and lack of performance orientation in compensation and appraisal are common characteristics in workgroups [53]. When guanxi HRM practice is prevalent in a workgroup, employees have the perception that guanxi typically influences supervisors’ HR decisions and that HRM practice is unfair to them [25]. Previous empirical studies have shown that even though employees accept the benefits of guanxi-influenced HRM decisions, they still perceive that procedural justice is damaged if others’ guanxi is stronger than their own [13]. Decreased fairness can result in distancing employees from their work, ultimately leading to low levels of occupational well-being [54].

On the other hand, guanxi HRM practice may deplete employees’ personal resources, such as certainty and perceived control. Employees may experience high uncertainty when they perceive that recruiting processes, task allocations, and performance appraisal are often decided based on guanxi rather than rules and regulations [25]. Supervisors used to employ good relationships to allocate rewards at work and hide corrupt transactions [55]. Employees must take extreme care so as not to damage the relationship with supervisors; they must apply supervisor-targeted impression management strategies such as pseudo-loyalty and gift-giving [11]. Even though employees who have good personal relationships with supervisors gain special privileges and favors, they may worry about being the victims of political intrigue [27]. Thus, guanxi HRM practice evokes anxiety, distress, and a sense of insecurity among team members. Employees lose control over external circumstances and feel that they have fewer individual rights, independence, and self-determination [56]. This kind of negative psychological feeling is associated with occupational well-being [57]. Based on the above reasoning, we expect that guanxi HRM practice tends to reduce job resources and personal resources to undermine occupational well-being.

**Hypothesis** **1.** *Guanxi HRM practice is negatively related to employees’ occupational well-being.*

From a COR theory perspective, guanxi HRM practice may trigger a spiral loss of psychological safety which may, in turn, harm employees’ occupational well-being at follow-up. Psychological safety is perceived as the freedom to show and employ oneself [58]. The COR theory states that the loss or potential loss of resources is psychologically threatening [59]. HRM practice plays an important role in building employees’ psychological safety because employees are particularly concerned about HR decisions [60]. Previous research has shown that psychological safety is a function of interpersonal factors such as trustworthy relationships and predictable leadership behaviors. Formal institutions or “rules of the game” offer an open, trustworthy, consistent situation for employees to feel secure and thus capable of changing their behavior [58]. By contrast, guanxi HRM practice, as a weak institutional environment, often creates an ineffective judicial system that provides poor protection for the workforce [13]. Hence, guanxi HRM practice, as a source of employees’ psychological distress, will directly affect employees’ perception of safety. In such a work environment, employees feel that their situations are insecure, unpredictable, and unclear in terms of behavioral consequences. They are afraid to speak honestly or to report a problem or mistake lest they lose the support of supervisors. There is now considerable evidence that members under guanxi HRM practice may experience additional pressure when their voice or behaviors could be interpreted as evidence for promotions, salary, or performance reviews [61]. Hence, guanxi HRM practice may threaten employees’ confidence and trust in the organization, jeopardize their self-expression, and eventually violate their psychological safety. Psychological safety as a personal resource is fundamental to individual identity and is a necessity for employee health in a demographically diverse workplace [62]. Reduced psychological safety leads employees to spend time and effort fraught with interpersonal risk (e.g., ridicule, neglect, and scolding) [63], which may consume cognitive and emotional resources that subsequently impair their well-being at work. When individuals cannot express their true selves, they tend to engage in the inauthenticity of faking expression, or surface acting from further damage [64], which threatens one’s sense of moral integrity and self-worth; therefore, occupational well-being decreases. Research has established that people who suppress their feelings and ideas experience more depletion [65]. Therefore, we can construct the following hypothesis:

**Hypothesis** **2.** *The negative relationship between guanxi HRM practice and occupational well-being is mediated by psychological safety.*

Due to the blurring of organizational boundaries and the proliferation of self-managing teams, team culture is increasingly important to HR practice [66]. People desire to appear culturally appropriate and good [67]. We make the point that collectivistic culture will exacerbate the negative effect of guanxi HRM practice on psychological safety for two main reasons. First, collectivistic culture promotes interdependence within the team, resulting in lower psychological safety. Under conditions of high team collectivism, interpersonal relationships are a key mechanism through which individuals become attached to workgroups; team members are more concerned about the appearance of risky interpersonal exchanges and more vigilant regarding interpersonal tensions [68]. In a highly collectivistic culture, employees devote more attention to contextual signs for building or maintaining relationships, meaning that guanxi HRM practice will be more operational for them, thus the perceived uncertainty and vulnerability in dealing with others on the team will deteriorate the negative effect of guanxi HRM practice on psychological safety. In cases where there is low team collectivism, maintaining interpersonal relationships is secondary [69]. Low collectivistic culture may alter the weight of guanxi HRM practice on psychological safety, as employees tend to protect their self-interests and may use means other than developing relationships to gain psychological safety. Thus, in a team lower in collectivism, a weaker relationship between guanxi HRM practice and psychological safety is expected. Second, collectivistic culture prescribes the values of ingroup obligation as well as obedience and loyalty to authority, maintaining that team members should be more tolerant of unethical treatment from supervisors [70], which aggravates the resource-loss process. In team cultures higher in collectivism, where the focus is on protecting harmonious relationships, employees feel compelled to comply with leaders’ decisions rather than retaliate against leaders’ disrespect and fairness violations [71]; it is unlikely that they will discipline a coworker who is favored under guanxi HRM practice. Thus, high collectivistic culture might indeed encourage the use of guanxi HRM practice to damage employees’ psychological safety. In contrast, in workgroups lower in collectivism, fairness is valuable to individuals, as they have a preoccupation with their rights and freedoms [72]. This indicates that employees will be more sensitive to the way they are treated and rewarded. When employees believe that there are guanxi HRM practices in the workgroup, they will have less tolerance for violating norms, fairness, and principles and place more emphasis on the protection of their resources, leading to a weaker relationship between guanxi HRM practice and psychological safety. Thus, we predict that:

**Hypothesis** **3.** *Team-level collectivistic culture moderates the relationship between guanxi HRM practice and psychological safety; the negative relationship is strengthened when collectivistic culture is higher and weakened when collectivistic culture is lower.*

## 3. Methodology

### 3.1. Sample and Procedure

Data were collected from two private firms and two state-owned companies in central China. We contacted the senior directors of these firms and introduced the research project. They showed interest in this project and agreed to take part in anticipation of receiving an overall report regarding the results of the study. We explained how this study would be carried out so that they could instruct their employees as to the details of this investigation. All respondents were assured that all information will be kept confidential and gave their informed consent electronically when the survey was carried out.

To minimize the potential for common method bias, the data were collected from multiple sources. Five hundred and eighty-eight employee participants were asked to respond to demographic questions and scales related to guanxi practice, psychological safety, and occupational well-being. A separate survey was employed to collect supervisors’ evaluations of the team’s collectivistic culture. After deleting missing data and matching employee data with team leader data, complete survey responses were available for 42 of the 55 teams invited (76.4 percent), with a total of 297 employees (50.5 percent). The employees’ average age was 32 years, and 57.5 percent were women. In total, 99 percent had completed a high school degree, and 69 percent held bachelor’s degrees or above.

### 3.2. Measurements

We used well-established scales to measure the constructs (e.g., guanxi HRM practice, psychological safety, occupational well-being, and collectivistic culture). Following the translation and back-translation procedure, we created a Chinese version of the scales for measuring these variables. All items in the study were rated on a 5-point Likert scale (1 = Strongly disagree to 5 = Strongly agree).

Guanxi HRM practice was measured with a 5-item scale of Chen et al. [8]. The respondents were asked to report their perceptions of the degree of HRM decisions. Items include “Many people joined my company through guanxi”, “Many people got promoted through guanxi”, “Bonuses and salary are often decided based on guanxi”, “Task allocations are often decided based on guanxi” and “Performance appraisals are often influenced by guanxi”. This scale has been successfully used in previous studies in mainland China. Ren and Chadee [61] tested the internal consistency of the scale in Chinese enterprises and found Cronbach’s alpha coefficient for guanxi HRM practice was 0.84. In the current study, the Cronbach’s alpha is 0.91. Considering that the guanxi HRM practice is nested in the workgroup approach to employee HRM practice, we tested for aggregation to the team level. We found support by significant between-group differences from the ANOVA test (F(41) = 1.88, *p* < 0.000), and high ICC (intraclass correlation coefficient) values (ICC 1 = 0.10; ICC 2 = 0.47) [73]. Thus, support was obtained for aggregating guanxi HRM practice to the team level.

Psychological safety was measured using the Edmondson [74] seven-item scale. The psychological safety scale has been shown to have convergent and divergent validity (e.g., [32]). The Cronbach’s alpha is 0.73. An example item is “if you make a mistake on this team, it is often held against you”.

Occupational well-being was measured using the Yvonne, Rodney and Kate [75] four-item scale. This scale has been used frequently in prior studies to measure well-being at work (see [46]). The Cronbach’s alpha is 0.83. An example item is “Overall, I think I am reasonably satisfied with my work life”.

Collectivistic culture was measured with a ten-item scale from Wagner and Moch [76]. The measurement describes cultural orientations and has been employed in prior studies to measure collectivistic culture (e.g., [70]). The Cronbach’s alpha is 0.88. An example item is “People in my work group should be willing to make sacrifices for the sake of the work group (such as working late now and then; going out of their way to help, etc.)”.

Following advice from previous studies (e.g., Warr [77], Doverspike and Blumenta [78]), we controlled for employees’ gender, age, education and tenure, as these might suggest their experience as they relate to their occupational well-being.

### 3.3. Preliminary Analyses

As participants in this study were working in 42 teams of four companies, it was possible that the nested data were non-independent. In consideration of that, we used the complex analysis in Mplus to control for the nested effects. We first conducted confirmatory factor analysis to examine the common method bias (CMB). Afterward, hierarchical multiple regression and Sobel test were applied to test hypotheses.

As the variables in the current study were self-reported, common method bias (CMB) might be a concern, and confirmatory factor analysis with Mplus 7.0 was conducted to check the problem of CMB. The results indicate that one-factor measurement model with Guanxi HRM, psychological safety, occupational well-being and collectivistic culture loaded on one general factor did not fit the data well (χ^2^/*df* = 5.91, RMSEA (root mean square error of approximation) = 0.13, SRMR (standardized root mean square residual) = 0.16, CFI (comparative fit index) = 0.40, TLI (Tucker–Lewis index) = 0.34), thereby suggesting that CMB is minimized in the current study. Based on the analyses mentioned above, the dataset was appropriate for further analysis.

## 4. Results

### 4.1. Descriptive Analysis

Table 1 summarized the means, standard deviations, and correlation coefficients with the corresponding significance level of studied variables. As recommended by Bernerth, Cole, Taylor, and Walker [64], we analyzed whether it was necessary to control for gender, age, education and tenure. The results show that these variables did not significantly relate to occupational well-being, so we did not control for these four socio-demographic variables when testing the hypotheses.

### 4.2. Hypothesis Testing

We employed multilevel regression analysis with Mplus 7.0 to test our hypotheses since the data included a group-level variable (e.g., guanxi HRM practice and collectivistic culture) and individual-level variables (e.g., psychological safety and occupational well-being). We tested our study hypotheses in three interlinked steps. First, we examined a model of guanxi HRM practice and occupational well-being (Hypothesis 1). Second, we examined a mediation model (Hypothesis 2). Third, we integrated the proposed moderator variable into the model and empirically tested the moderation model (Hypothesis 3).

Hypothesis 1 posited that guanxi HRM practice was negatively related to employees’ occupational well-being. In Model 1 of Table 2, the results suggest that guanxi HRM practice was negatively related to employees’ occupational well-being (*γ* = −0.427, SE = 0.089, *p* < 0.001), supporting Hypothesis 1.

Hypothesis 2 stated employee psychological safety mediated the relationship between guanxi practice and employees’ occupational well-being. Model 2 showed that when psychological safety was included, the link between psychological safety and occupational well-being was significantly positive (γ = −0.318, SE = 0.062, *p* < 0.001), while the direct path from guanxi HRM practice to occupational well-being became less significant (γ = −0.266, SE = 0.127, *p* < 0.05), as shown in Model 2. Meanwhile, guanxi HRM practice was negatively associated with psychological safety, as shown in Model 3 (γ = −0.323, SE = 0.06, *p* < 0.001), indicating a partial mediation. Hence, Hypothesis 2 was preliminarily supported. To further test Hypothesis 2, Sobel’s [79] test was suggested as a significance test for the indirect effect. The Sobel test was strongly significant (Sobel test statistic = 3.77, *p* < 0.001), suggesting that psychological safety partially mediates the main effects of guanxi HRM practice on employees’ occupational well-being, thus providing support for Hypothesis 2.

**Figure 2:**
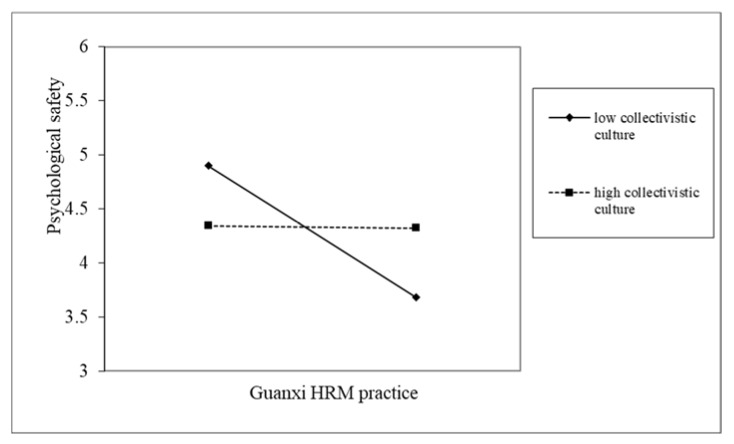
Interactive effects of guanxi HRM practice and collectivistic culture on psychological safety.

Hypothesis 3 assumed that team collectivistic culture positively moderates the relationship between guanxi HRM practice and psychological safety. As indicated by Model 4 of Table 2, the cross-level interaction coefficient of “collectivistic culture × guanxi HRM practice” is significantly related to employee psychological safety (*γ* = 0.30, SE = 0.147, *p* < 0.05). This finding suggests that collectivistic culture positively moderates the relationship between guanxi HRM practice and psychological safety and supports Hypothesis 3. Following Aiken and West’s [80] methods, we plotted this interactive effect, as shown in Figure 2. As Figure 2 reveals, the relationship between guanxi HRM practice and psychological safety was more pronounced at high levels of collectivistic culture than at low levels of collectivistic culture. Therefore, Hypothesis 3 was further supported.

## 5. Discussion

There has been growing interest in understanding the impact of guanxi HRM practice on employee outcomes (e.g., Ko and Liu [81]). The primary goal of this study was to expand this field of research by examining how guanxi HRM practice affects employees’ occupational well-being. Using data from 297 employees on 42 teams from four companies, our results fully supported our hypotheses. As predicted, guanxi HRM practice negatively affected employees’ perception of psychological safety, which, in turn, impacted their occupational well-being. In addition, the effect of guanxi HRM practice on psychological safety was stronger when group collectivism was high or when collectivism was low. We discuss the implications of these findings.

### 5.1. Theoretical implications

This study contributes to the literature in several ways. First, drawing on the COR theory, we investigated whether and how guanxi HRM practice affects employees’ occupational well-being. The present study is meaningful because most research regarding HRM has focused on the financial return [44,82]. Furthermore, this research enhances our knowledge of the negative effects of guanxi HRM practice, which is widely implemented in Chinese organizations. Two competing views stand out in the literature with respect to the relationship between HRM and employee well-being. From a “mutual gains” perspective, HRM has positive effects both on employee well-being and performance [83]. From a “conflicting outcomes” perspective, HRM pays off in terms of employee performance but has a detrimental impact on employee well-being [84]. Our findings highlight resource depletion as a promising explanation of how guanxi HRM practice influences employees’ occupational well-being. This finding makes a connection to the “dark side” of HRM and responds to recent calls for research exploring the negative aspects of HRM in order to provide a fuller and more meaningful understanding of HRM [85].

Second, our study further addresses the question of why guanxi HRM practice relates to employees’ occupational well-being by outlining a plausible mediating mechanism, such as psychological safety. Gu, Nolan, and Rowley’s [86] study of the relationship between guanxi HRM practice and performance appraisal identifies perceptions of fairness as an intervening process. We further extend the literature by adopting a psychological safety perspective based on the COR view of occupational well-being. Psychological safety is an important mediator from a COR perspective, which suggests that employees who experience an uncontrollable and unpredictable work context are likely to experience low psychological safety, which in turn reduces their occupational well-being. Supporting this argument, previous studies have shown that lower psychological safety is caused by supervision incivility, unsupportive work context [87], and status conflict [88]. Guanxi HRM practice, as a hostile interpersonal environment, may deteriorate team members’ perceptions of psychological safety. For the development of occupational well-being, drawing on the COR theory to identify unique mechanisms translating guanxi HRM practice into various employee outcomes will be genuinely intriguing.

Third, by isolating a potential boundary condition for guanxi HRM practice, we further offer an elaborate understanding of when guanxi HRM practice impedes psychological safety. Recently, COR theorists have begun to search for potential contextual factors that may constrain or facilitate the conservation of resources process [89]. Although psychological safety is related to social interaction, it is unclear how team cultural factors shape processes related to psychological safety. Edmondson and Lei [58] called for future research into this issue by examining different cultural contexts in which “employees may be particularly hesitant to ask questions, provide feedback, or openly disagree with their superiors” (p. 8). Our research suggests that collectivistic culture exacerbates the deleterious effect of guanxi HRM practice on psychological safety. Employees in a highly collectivistic culture tend to maintain harmonious interpersonal relationships during the application of guanxi HRM practice, and guanxi HRM practice should be more harmful to their psychological safety. By theoretically framing collectivistic culture as a moderating contingency, this study further contributes to the mixed findings related to the main effect of guanxi HRM practice (e.g., Yang [23]). Doing so could help better understand the resource depletion processes triggered by guanxi HRM practice.

### 5.2. Practical Implications

From a practical perspective, the present study provides meaningful messages for HR managers. Occupational health psychology aims to create a psychologically healthy and safe workplace to promote workers’ well-being and prevent harm to their health [90]. Considering that guanxi HRM practice runs the risk of decreased occupational well-being by creating a psychologically unsafe environment, managers should try to limit guanxi HRM practice. As Hsu and Wang [91] noted, guanxi HRM practice as an “unnecessary evil” blocks employee development. For example, leaders should make human resource decisions according to merit or ability rather than guanxi. Accordingly, the HR decisions among members become relatively clear and stabilized, and members may recognize the work performance norms, leading to greater perceived occupational well-being.

Second, our results suggest that the effect of guanxi HRM practice on employees’ occupational well-being is mediated by psychological safety. Thus, when guanxi HRM practice has previously occurred and the members have become highly psychologically unsafe, it might be necessary to build a psychologically safe climate to enhance occupational well-being. For example, leaders can work to create practices such as shared goals, shared knowledge, job autonomy, and mutual respect to foster psychological safety and attempt to maintain fair and socially favorable interactions, as well as creating a supportive work environment in which employees may discharge any insecurities and anxieties.

Finally, our findings regarding the exacerbating impact of guanxi HRM practice under highly collectivistic culture suggest that organizations should not build collectivistic cultures to deal with the ill effects of guanxi HRM practice; in fact, employees place a premium on maintaining relationships within a context in which guanxi HRM practice interacts with collectivistic culture. Indeed, managers should create a supportive diversity climate conveying a positive message to all employees, in which employees place more emphasis on uniqueness and norms [92], which in turn inspire psychological safety among employees.

## 6. Limitations and Future Research

Despite these important contributions, several limitations warrant further discussion and future research. First, the present finding may be contaminated by common method variance, as the data related to guanxi HRM practice, psychological safety, and occupational well-being came from the same source. However, the assessment of collectivistic culture was performed by immediate supervisors, which helps to reduce potential issues with common method bias [93]. Second, given the use of cross-sectional data, this study was not able to definitively establish causal inference regarding the relationships. There might be other alternative mediating factors that could explain the relationship between guanxi HRM practice and occupational well-being. Future research might conduct longitudinal and experimental designs to examine these mechanisms more closely. Third, this study adopted a general occupational well-being measure, although some researchers divided employee well-being into six dimensions [94]. Guanxi HRM practice and psychological safety may be related to these six dimensions in different ways. Future research is needed to examine the effects of guanxi HRM practice on the six dimensions of occupational well-being. Finally, we have only examined one boundary condition of the link between guanxi HRM practice and psychological safety, namely, collectivistic culture. Future research can examine whether other dimensions of organizational culture might moderate the effects of guanxi HRM practice on employee outcomes.

## 7. Conclusions

This study explored the negative effects of guanxi HRM practice on employees’ occupational well-being and tested the mediating role of psychological safety and the moderating role of collectivistic culture. Our results provide important theoretical and practical implications for employee well-being and guanxi HRM practice. Future studies on employee well-being should expand the existing COR theory, which focuses on the relationship between HRM and employee outcomes. Moreover, future implementation efforts must consider improving the occupational well-being of employees and enhance their psychological safety to reduce the negative impact of guanxi HRM practice on health-related productivity.

## Figures and Tables

**Table 1:** Means (M), standard deviations (SD) and correlation coefficients.

Variables	Mean	SD	1	2	3
Individual level (*n* = 297)					
1. Psychological safety	3.19	0.47			
2. Occupational well-being	3.49	0.64	0.47 **		
Team level (*n* = 42)					
3. Guanxi HRM practice	3.23	0.42	−0.29 **	−0.28 **	
4.Collectivistic culture	3.94	0.40	−0.03	0.05	0.06

Notes: ** *p* < 0.01.

**Table 2:** Regression results for main, mediation and moderation effects.

Variables	Occupational Well-Being		Psychological Safety	
	M1	M2	M3	M4
Independent Variable (level 2)				
Guanxi HRM practice	−0.427 ***(0.089)	−0.266 *(0.127)	−0.323 ***(0.060)	−0.305 ***(0.051)
Mediator (level 1)				
Psychological safety		−0.318 ***(0.062)		
Moderator (level 2)				
Collectivistic culture				0.015 (0.052)
Interaction (level 2)				
Collectivistic culture * guanxi HRM practice				0.300 *(0.147)

Notes: level 1: individual level, level 2: team level; *N*_individual_ = 297, *N*_team_ = 42. * *p* < 0.05; *** *p*< 0.001.
